# Elevated microRNA-125b inhibits cytotrophoblast invasion and impairs endothelial cell function in preeclampsia

**DOI:** 10.1038/s41420-020-0269-0

**Published:** 2020-05-13

**Authors:** Qinghua Li, Yangyang Han, Peng Xu, Lingxuan Yin, Yanru Si, Cuijuan Zhang, Yuhan Meng, Weiguo Feng, Zhifang Pan, Zhiqin Gao, Jie Li, Weiwei Yang

**Affiliations:** 1 School of Public Health, Weifang Medical University, Weifang, 261053 Shandong China; 2 School of Biosciences, Weifang Medical University, Weifang, 261053 Shandong China; 3 Shandong Province Key Laboratory of Biopharmaceutics, Weifang, 261053 Shandong China; 4 School of Life Science, Shanxi University, Taiyuan, 030006 Shanxi China; 5 Department of Obstetrics, Affiliated Hospital of Weifang Medical University, Weifang, 261031 Shandong China; 6 Center for Reproductive Medicine, Affiliated Hospital of Weifang Medical University, Weifang, 261042 Shandong China

**Keywords:** Pre-eclampsia, Epigenetics

## Abstract

Preeclampsia (PE) is a life-threatening disorder of human pregnancy affecting 5–8% of all pregnancies. Currently, PE remains an elusive complicated and heterogenous medical condition with no early marker or symptoms is recognized for this serious pregnancy complications. Here, we profiled the plasma miRNA expression patterns associated with preeclampsia and found 16 miRNAs were deregulated (*p* < 0.01) in patients who later developed PE. Circulating hsa-miR-125b was aberrantly upregulated in early pregnancy and significantly reduced after delivery in preeclampsia. We then investigated the underlying molecular mechanisms between miR-125b and PE in vitro. We found that upregulated miR-125b can target KCNA1 to inhibit trophoblast invasion in human trophoblast cells. Moreover, overexpression of miR-125b in HUVECs impaired endothelial cell function through GPC1. The findings indicated that upregulated miR-125b leads to impaired placentation, and an increased risk of preeclampsia, Our studies provide novel insights into the underlying mechanisms on the association of miR-125b in early pregnancy and risk of PE, miR-125b might be a more specific predictive marker and a safe therapeutic target for treating patients with PE.

## Introduction

Preeclampsia (PE) is a complicated syndrome of human pregnancy characterized by new occurred hypertension and signs of damage to another organ system, most often the liver and kidneys, after the 20^th^ week of gestation^1,2^. PE was diagnosed in at least 5–8% of all pregnancies and attributed to 76,000 and 500,000 deaths of maternal and fetuses worldwide, respectively^3^. It is widely recognized that PE origins with an asymptomatic phase featured by defects in trophoblast invasion and spiral artery remodeling during early pregnancy, which results in abnormal placentation, leading to placental ischemia and maternal systemic syndrome of PE in the later phase of gestation^4^. Despite extensive research by physicians and researchers, PE remains an elusive complicated and heterogenous medical condition, no early marker or symptoms is recognized and there is no effective medical treatment for this serious pregnancy complications.

MicroRNAs (miRNAs) are endogenous, short, noncoding RNA molecules that exert post-transcriptional regulation by repressing the translation and/or promoting degradation of target mRNAs^5,6^. miRNAs have been demonstrated in the modulation of all the cellular functions that have been studied so far and could serve as potential biomarkers for the early detection of various disease^7^. Accumulating reports have shown the differential miRNAs expression patterns in the term placenta and circulation of pregnant women suffering from PE^8–12^. Deregulated miRNAs were reported to participate in all fundamental aspects of placentation, including trophoblast proliferation^13–15^, differentiation^16,17^ and invasion^18–20^, endothelial cell function^21,22^, and inflammatory response^23–25^. However, most of these researches are limited to measure these miRNAs after the disease onset or at the time of delivery. Little is known about the circulating miRNAs patterns in early pregnancy.

In the present study, we hypothesized that the miRNAs expression signature would be different in early pregnancy. We compared the plasma miRNA expression pattern in the late first trimester between preeclampsia and healthy control pregnancies and found an aberrantly upregulated circulating miR-125b in early pregnancy which subsequently reduced after delivery in preeclampsia. We found that upregulated miR-125b can target KCNA1 to inhibit trophoblast invasion in human trophoblast cells. Moreover, overexpression of miR-125b in HUVECs impaired endothelial cell function through GPC1. Our results provided novel clues about the molecular mechanisms underlying the association between maternal miR-125b in early pregnancy and future risk of PE, suggesting miR-125b as a potential predictive marker and treatment target for PE.

## Results

### Early pregnancy miR-125b status was associated with the risk of PE

**Fig. 1:**
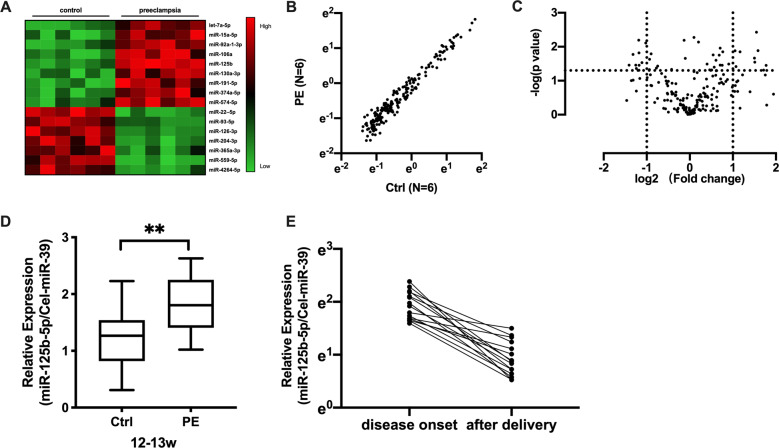
Early pregnancy miR-125b status was associated with risk of PE. **a** Heatmap of top plasma miRNA candidates upregulated in the plasma between PE (*n* = 6) and control pregnancies (*n* = 6). Red indicates upregulation and green indicates downregulation of miRNA level. 16 miRNAs were identified as deregulated in the plasma of patients who went on to develop PE by microarray. Scatter plots (**b**) and volcano plot (**c**) for preeclampsia and control comparisons using average miRNAs expression data were presented. **d** miR-125b expression in PE and control plasma. Blood samples between 12 + 0 and 13 + 6 weeks of gestation were collected when pregnant women went for their initial hospital appointment. miR-125b was significantly elevated in patients who finally developed PE (*n* = 15) compared to control (Ctrl, *n* = 29). **p < 0.01. **e** Comparison of plasma miR-125b before and 48 h after delivery. Blood samples were collected when preeclampsia patients went hospital due to disease onset and after the delivery for 1 week. All data are represented as mean ± SEM, asterisks indicate significance, with *p* < 0.01 by Student’s *t* test.

We conducted a miRNA microarray assay using samples from PE and gestation age-matched control samples in a nested case-control study. After the raw data were normalized, we identified 16 miRNAs were deregulated in the 12th–13th week between the preeclampsia and control group (Fig. 1a), of which nine miRNAs were significantly upregulated (fold change ≥2.0) and seven miRNAs were significantly downregulated (fold change ≤0.5) in the circulation of pregnancies who later developed preeclampsia (Table 1). As shown in the scatter plot (Fig. 1b) and volcano plot (Fig. 1c), we observed the miRNAs expression profiles were obviously different between preeclampsia patients and healthy control.

**Table 1:** Circulating miRNAs were deregulated in patients who went on to preeclampsia compared with normal early pregnancies.

miRNA name	Fold change	*P* value
let-7a-5p	2.5392	0.02481
miR-15a-5p	3.2710	0.00467
miR-92a-1-3p	2.8989	0.01597
miR-106a	3.0611	0.02155
miR-125b	3.8660	0.00498
miR-130a-3p	2.7468	0.04843
miR-191-5p	3.7475	0.02196
miR-374a-5p	3.2774	0.01547
miR-574-5p	2.1318	0.01255
miR-22–5p	0.3301	0.04533
miR-93-5p	0.2917	0.00839
miR-126-3p	0.4374	0.04285
miR-204-3p	0.2994	0.03887
miR-365a-3p	0.4652	0.01979
miR-559-5p	0.3351	0.02102
miR-4264-5p	0.3934	0.03123

We first validated circulating levels of miR-125b in a group of 15 patients who developed PE later and 29 gestational age-matched control. We found that, in early pregnancy (12th to 13th week of gestation), expression levels of miR-125b was significantly upregulated in patients who went on to develop PE compared to control pregnancies (Fig. 1d). ROC assay (Fig. S1D) revealed the diagnostic performance of plasma miR-125b that plasma miR-125b in early pregnancy had a high accuracy in identifying PE patients before disease onset with an AUC of 0.7632 ± 0.0743 (95% CI, 0.6177–0.9088, *p* < 0.01). To our great interest, the plasma level of miR-125b was still upregulated when disease onset but significantly reduced after delivery (Fig. 1e), implying that plasma miR-125b could serve as a potential early pregnancy biomarker of the risk of PE.

### Downregulation of KCNA1 and GPC1 were linked to upregulated miR-125b in preeclampsia

**Fig. 2:**
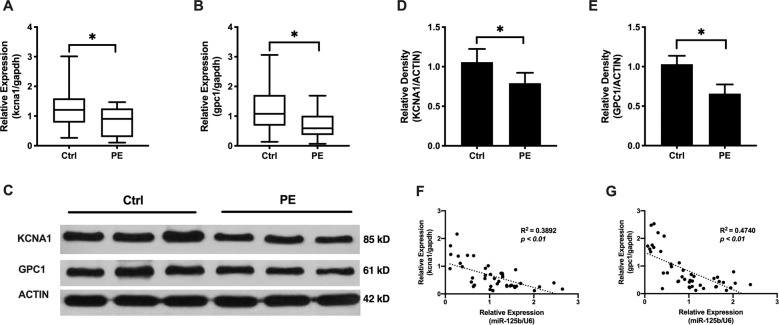
KCNA1 and GPC1 were downregulated in the placenta of PE patients. **a**, **b** mRNA expression of KCNA1 (**a**) and GPC1 (**b**) in placentas derived from PE patients (*n* = 15) and matched control (Ctrl, *n* = 29) were detected by real-time qPCR. **c** Western blot was carried out to measure protein expression of KCNA1 and GPC1 in the placenta of PE patients. Image J was used to quantify relative density. Statistical analysis of protein expression of KCNA1 (**d**) and GPC1 (**e**) in placentas derived from PE patients (*n* = 15) and matched control (Ctrl, *n* = 29) were presented using Graphpad Prism 7.0. The inverse correlation between miR-125b and KCNA1 (**f**), GPC1 (**g**) expression in the placenta of studied individuals is shown. All data are represented as mean ± SEM, *p < 0.05.

To determine which genes are downstream of miR-125b, we identified putative targets using miRNA-target prediction algorithms (Targetscan, miRanda, MirTarget). miR-125b was predicted to target KCNA1 and GPC1 mRNA transcripts. We first compared the levels of KCNA1 and GPC1 in PE placentas using real-time qPCR and western blot. Compared to gestational age-matched placentas from pregnancies without other clinical symptoms, the mRNA levels of KCNA1 and GPC1 were significantly reduced ~51% and 35% in PE placentas, respectively (Fig. 2a, b), and the protein levels of KCNA1 and GPC1 were decreased around 30% and 50% in PE samples, respectively (Fig. 2c–e). Further Pearson’s correlation analysis suggested miR-125b was reversely correlated with KCNA1 (*R*^*2*^ = 0.3892 and *p* < 0.01, Fig. 2f) and GPC1 (*R*^*2*^ = 0.4740 and *p* < 0.01, Fig. 2g) in placentas. These data indicated a promising role of KCNA1 and GPC1 in PE.

### Localization with KCNA1 and GPC1 in human placenta

**Fig. 3:**
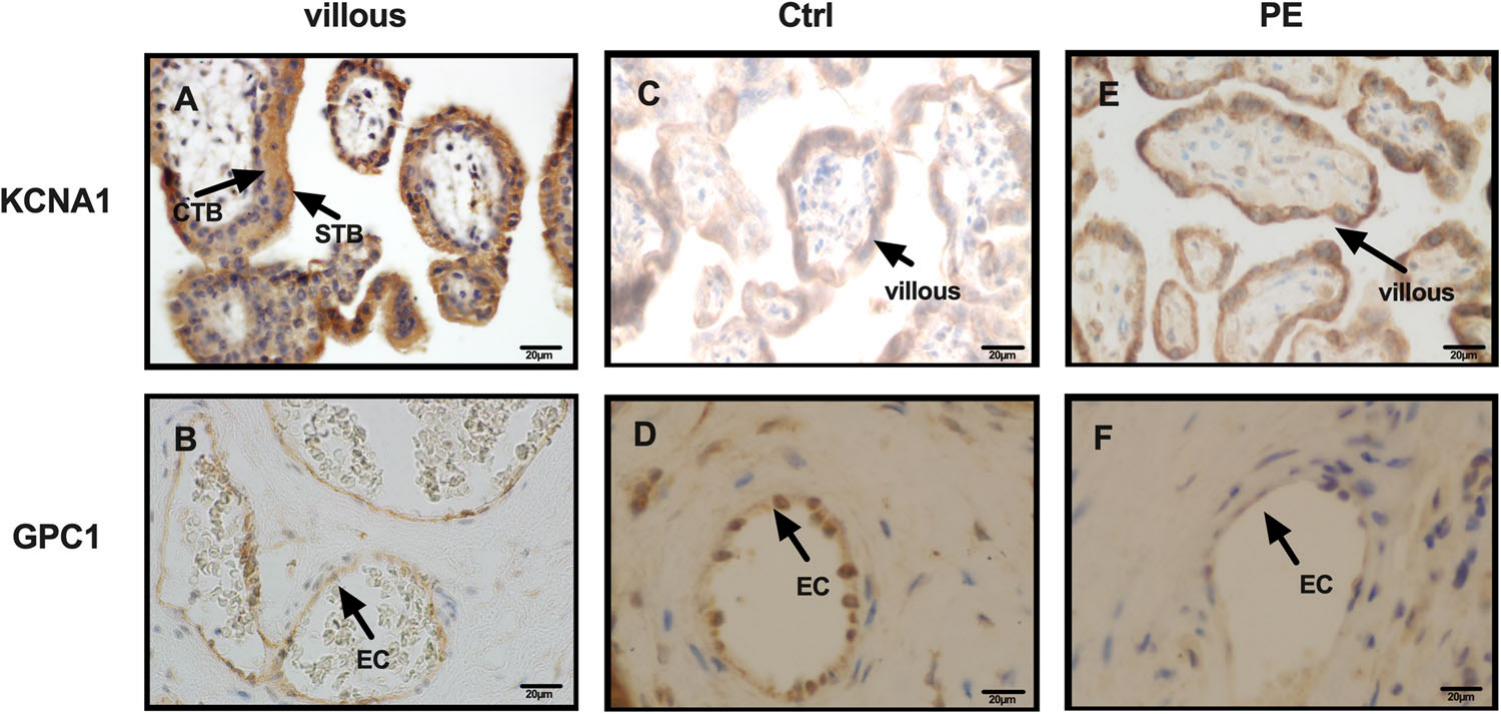
KCNA1 and GPC1 were localized in human placenta villi. Immunohistochemistry was performed on paraffin sections of human placenta villi (**a**, **b**) at early gestation and preeclamptic placenta at term placenta (**c**–**f**). At early pregnancy, the positive immunoreactivity for KCNA1 was mainly located in various trophoblast cells, including cytotrophoblast cells and syncytiotrophoblast cells (**a**), while GPC1 was predominantly observed in vascular endothelial cells (**b**). At term placenta, KCNA1 remained in various trophoblast cells (**c**–**e**), while GPC1 mainly located in vascular endothelial cells (**d**–**f**). CTB cytotrophoblast cells, EVT extravillous trophoblast cells, EC vascular endothelial cells.

We then examined the spatial expression profile of KCNA1 and GPC1 in the placenta villi using immunohistochemistry. We observed that KCNA1 mostly localized in trophoblast cells, especially cytotrophoblast cells and extravillous trophoblast cells (Fig. 3a), and GPC1 predominantly observed in vascular endothelial cells (Fig. 3b). The immunostaining of KCNA1 was intensively limited to trophoblast cells in PE (Fig. 3c–e) while positive signals of GPC1 were primarily found in vascular endothelial cells (Figs. 3d–f and S7). The localization pattern suggested their promising participation in the placentation, KCNA1 could function in the regulation of trophoblast invasion and GPC1 may exert its effect in angiogenesis.

### KCNA1 and GPC1 were direct targets of miR-125b in the placenta

We next evaluated the potential effect of miR-125b on KCNA1 and GPC1 expression in the human placental cells. We first transfected trophoblast cells and human umbilical vascular endothelial cells (HUVECs) with miR-125b or control miRs and monitored changes in their mRNA and protein expression. Parallel dishes were transfected with corresponding control miRs as the negative control.

**Fig. 4:**
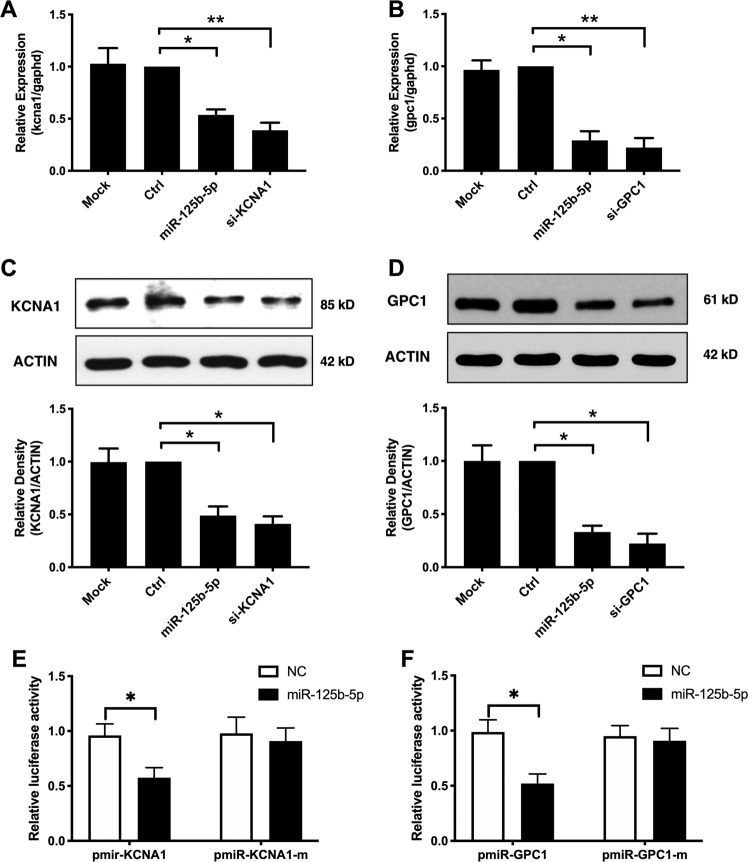
KCNA1 and GPC1 were validated to be direct targets of miR-125b. The human trophoblast cells, HTR8/SVneo, were transfected with miR-125b mimics or negative control miRs. mRNA levels (**a**) and protein levels (**c**) of putative target KCNA1 was evaluated post transfection using real-time qPCR and western blot separately. Data are presented as mean ± SEM of four independent experiments in triplicate. *p < 0.05, **p < 0.01. The human endothelial cells, HUVECs, were transfected with miR-125b mimics or negative control miRs. In total, 48 h later, mRNA levels (**b**) and protein levels (**d**) of putative target KCNA1 was evaluated using real-time qPCR and western blot separately. Data are presented as mean ± SEM, *N* = 4 performed in triplicate. *p < 0.05, **p < 0.01. **e**, **f** HEK-293T cells were co-transfected with luciferase reporter construct and miR-125b mimics. Luciferase activities were determined 24 h post transfection. Data are presented as mean ± SEM values of four independent experiments, each conducted in triplicate. *p < 0.05.

As presented in Fig. 4a, mRNA expression of KCNA1 was significantly reduced in human trophoblast HTR8/SVneo cells after miR-125b transfection for 48 h, similar to KCNA1 knockdown. Immunoblot analysis also indicated that expression KCNA1 was significantly reduced 48 h post-transfection of miR-125b mimic (Fig. 4c). In contrast, inhibition of endogenous miR-125b by miR-125b inhibitor significantly upregulated KCNA1 expression (Fig. S2). The data demonstrated that KCNA1 was significantly inhibited by miR-125b in human trophoblast cells.

The mRNA and protein expression of GPC1 was significantly suppressed in HUVECs that were transiently transfected with miR-125b (Fig. 4b–d). Conversely, transfection of HUVEC cells with miR-125b inhibitor significantly increased GPC1 expression (Fig. S3). The finding that the transcripts and protein were inversely regulated indicates that GPC1 appear to be targets of miR-125b in HUVEC cells.

Putative miR-125b binding sites within the 3′-UTRs of KCNA1 and GPC1 were identified using the target prediction tool TargetScan^26^. The 3′-UTRs of KCNA1 and GPC1 each contain one miR-125b specific binding site (Fig. S4A). We performed dual-luciferase reporter assays to validate the direct targeting effect of KCNA1 and GPC1 by miR-125b. HEK-293T cells were co-transfected with miR-125b and with pmirGL3 reporter plasmids in which the 3′-UTRs of KCNA1 and GPC1 transcripts with (mutated) or without (wild-type) point mutations in the miR-125b binding site (Fig. S4B). As shown in Fig. 4e, f, for each of the wild-type vectors, miR-125b mimics induced a significant reduction in luciferase activity. However, co-transfection of miR-125b with luciferase vectors of mutated 3′-UTR vectors did not affect the luciferase activity. Taken together, we concluded that KCNA1 and GPC1 were directly targeting by miR-125b.

### miR-125b impairs trophoblast invasion and disturbs endothelial cell function

**Fig. 5:**
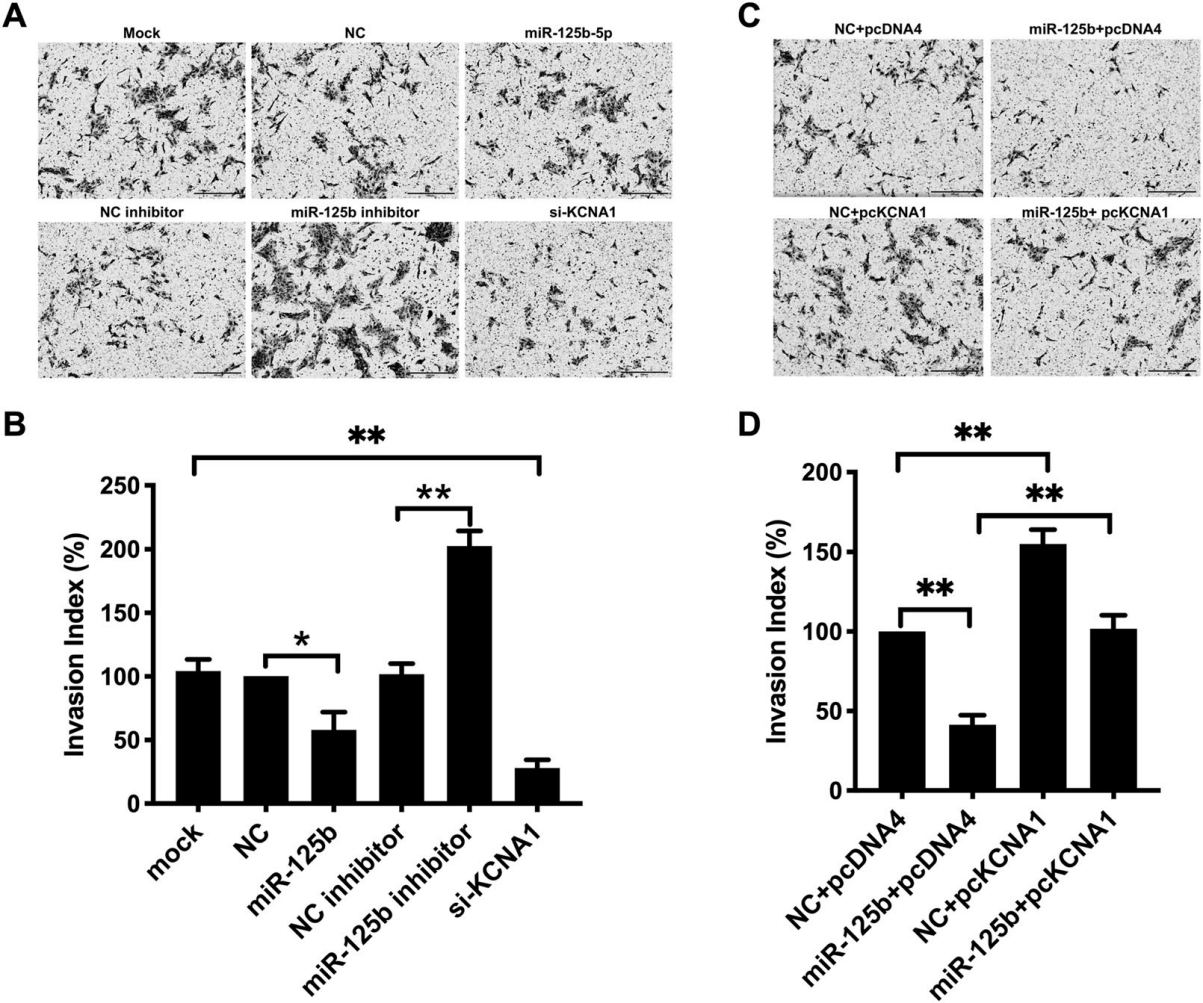
miR-125b inhibited trophoblast invasion via KCNA1 mediation. The invasiveness of HTR8/SVneo cells transfected with miR-125b, miR-125b inhibitor, and KCNA1 siRNA were determined using Transwell insert invasion assay, as presented the typical field in **a** and relative invasion index in **b**. *N* = 3 performed in triplicates and results are presented as mean ± SEM. Statistical comparison between miR-125b mimics (or inhibitor) and the negative control was performed using Student *t* test, with *p* < 0.05 considered as significant. *p < 0.05, **p < 0.01. **c** Typical microscope fields of Transwell insert invasion assays to examine cell invasion in HTR8/SVneo cells transfected with either KCNA1 overexpressing pcDNA 4 vector (pcKCNA1) or pcDNA 4 vector (pDNA4). miR-125b mimic or negative control miRs was co-transfected. **d** Relative invasion index based on 3 independent experiments performed in triplicates and results are presented as mean ± SEM. with *p* < 0.05 considered as significant. *p < 0.05, **p < 0.01.

Impaired trophoblast invasion is an early critical event in PE. KCNA1 was reported to modulate the invasiveness in tumor cells^27,28^. Here, we estimated the effect of miR-125b in modulating the invasiveness of trophoblast cells. To gain insights into the regulation of miR-125b on trophoblast invasion, we transfected HTR8/SVneo cells with miR-125b mimics or inhibitor. We observed that miR-125b significantly inhibited trophoblast invasion by 58.6%, while repression of endogenous miR-125b with specific miR-125b inhibitor had opposite effects (Fig. 5a, b). We further assessed whether KCNA1 participated in the invasion-inhibitory effect of miR-125b in human trophoblast cells via co-transfection of miR-125b and KCNA1 overexpression vector (Fig. S5), we found that overexpression of KCNA1 gene significantly abolished the invasion-inhibitory effect of miR-125b (Fig. 5c, d). The data demonstrated that miR-125b significantly inhibited trophoblast invasion via KCNA1.

**Fig. 6:**
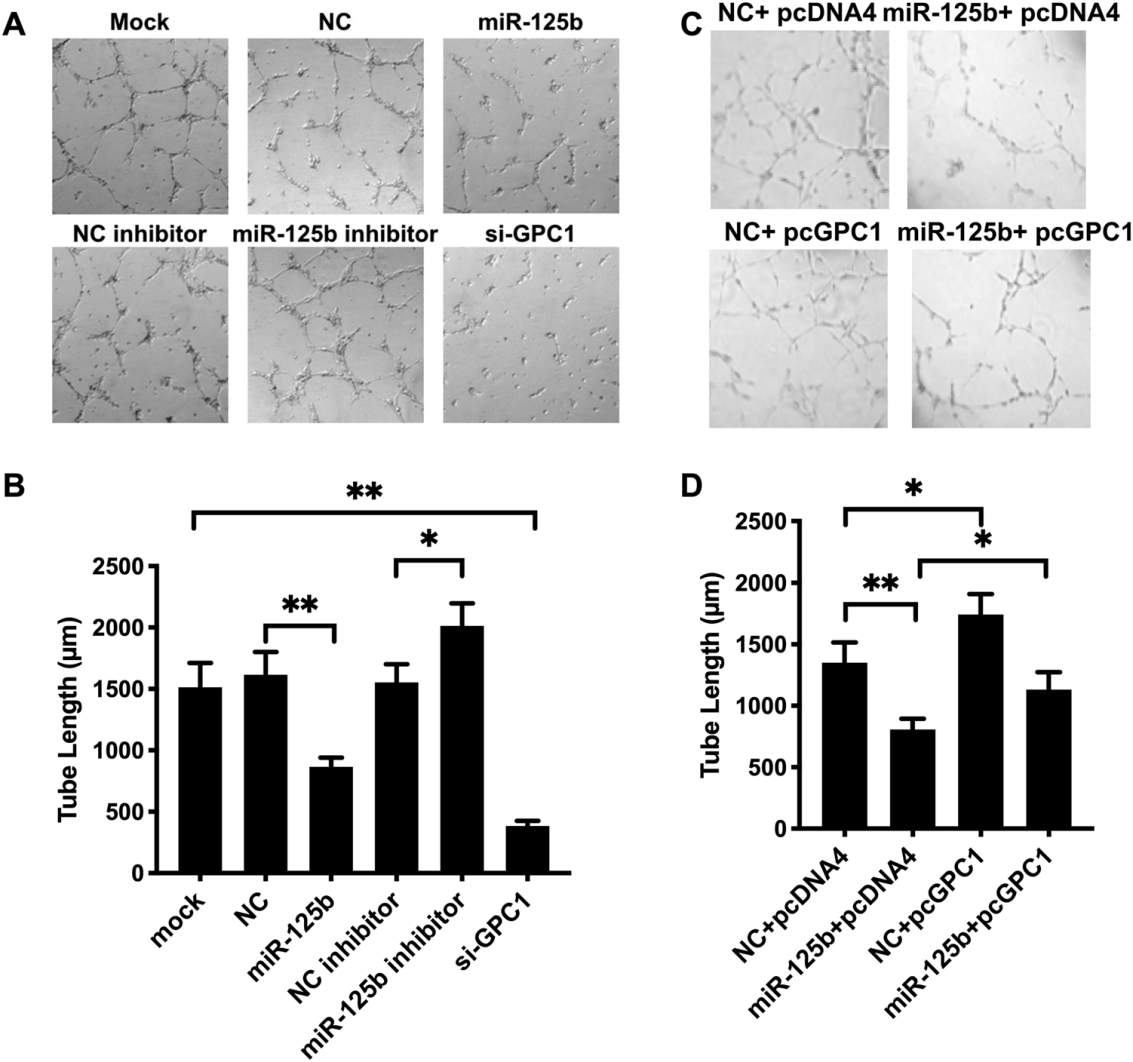
miR-125b impaired trophoblast invasion through GPC1. HUVECs were transfected with transfected with miR-125b, miR-125b inhibitor, or GPC1 siRNA, and then cultured in the Matrigel-coated plates (BD Bioscience). At 6 h after seeding, images were collected from five random fildes per well using light microscopy. The total tube length was measured and analyzed using Image J, as shown the typical field in **a** and tube length in **b**. *N* = 4 conducted in triplicates and results are presented as mean ± SEM, with *p* < 0.05 considered as significant. *p < 0.05, **p < 0.01. **c** Typical microscope fields of tube formation assays in HUVECs transfected with either GPC1 overexpressing pcDNA 4 vector (pcGPC1) or pcDNA 4 vector (pDNA4). miR-125b mimic or negative control was co-transfected. **d** Tube length were based on four independent experiments carried out in triplicates and results are presented as mean ± SEM. with *p* < 0.05 considered as significant. *p < 0.05, **p < 0.01.

Defective angiogenesis of the placenta is another key factor in PE development. Upregulation of miR-125b in HUVECs suppressed expression of GPC1, which was crucial for angiogenic potential in different cells^29,30^. Therefore, we addressed whether increased miR-125b engaged in the regulation of the angiogenic potential of endothelial cells in vitro. We conducted a tube formation assay with HUVECs transfected with miR-125b. As presented in Fig. 6a, b, HUVECs transfected with miR-125b formed fewer tubes than the negative control cells, importantly, inhibition of the endogenous miR-125b increase tube formation of HUVECs. Rescue experiments also demonstrated that the upregulation of GPC1 (Fig. S6) significantly ameliorated the inhibitory effect of miR-125b on tube formation, which further evidenced the mediating effect of GPC1 in the miR-125b-inhibitory effect of angiogenesis (Fig. 6c, d). These results showed that miR-125b regulated endothelial cell angiogenic functions through GPC1.

## Discussion

Here we reported that upregulated miR-125b in early pregnancy was associated with the risk of PE and identified its biological and pathological relevance. In early pregnancy, the circulating level of miR-125b was significantly upregulated in early pregnancy in patients who went on to develop PE, and the upregulation of miR-125b continued on until term and reduced after the delivery. We found that KCNA1 and GPC1 were downregulated in placentas derived from PE patients, miR-125b inversely regulated the expression of KCNA1 in trophoblast cell and GPC1 in endothelial cells. Luciferase assays validated that KCNA1 and GPC1 are directly targeted by miR-125b. We further observed that upregulated miR-125b inhibited trophoblast invasion via KCNA1, and transfection of miR-125b suppressed endothelial function through GPC1. Our results demonstrated that the upregulation of miR-125b resulted in inhibited trophoblast invasion and endothelial dysfunction in placentation during early pregnancy, thus impaired placentation leading to maternal systemic pathological phenotypes of PE.

The impetus for the present study is the growing interest regarding the early identification of preeclampsia patients using circulating deregulated miRNAs^31–34^. Characterization of prospective plasma miRNAs signature at the early gestation stage would provide a valuable insight into pathological factors of the syndrome and benefit the development of new early diagnostic biomarkers and potential targets for therapeutic intervention. Deregulated miRNAs have been shown to regulate signaling pathways participated in trophoblast cell migration/invasion and endothelial cell function at the interface between maternal and fetus, thus impacting on placental and maternal phenotypes^8^. However, most of the studies measured miRNAs in samples from third pregnancy after the clinical symptoms manifest or at delivery while the key pathophysiological changes started before 20th weeks of gestation^35^. There is a paucity of data that has assessed the miRNAs expression patterns in early pregnancy. Therefore, in this study, we hypothesized that deregulated miRNAs expression in early pregnancy contributed to the pathogenesis of preeclampsia.

Within this overall hypothesis, we first profiled the miRNAs expression in early pregnancy. We carried out a nested case–control study to assess miRNAs signature in plasma using miRNA microarray assay. The plasma samples were collected from early pregnancy (12th to 13th week of gestation) when patients visit the hospital for their first appointment. Our results showed that plasma miRNAs were deregulated in early pregnancy of patients who later developed PE. The circulating miR-125b was validated to significantly upregulated before symptoms. The upregulated miR-125b was also observed when the syndrome diagnosed, however, expression levels of miR-125b rapidly returned to nearly normal levels after the delivery, thus evidencing a close relationship with the adverse disorder. Collectively, the findings strongly indicated miR-125b participation in the early pathological changes of preeclampsia.

Because we found such a potential miRNA in plasma during early pregnancy, one important question to be addressed is whether miR-125b contribute to the early pathological events of preeclampsia. miR-125b is a highly conserved miRNA across several hemochorial placenta species (Fig. S1A), miR-125b has been recognized for its role in various cancers with its diverse function to promote or suppress carcinogenesis^36^, miR-125b also exhibited significant effects on various phenotypes of endothelial cells and vascular smooth muscle cells during vascular diseases^37^. Recent studies demonstrated miR-125b was upregulated in preeclamptic placentas^23^ and could be exported from human placenta into maternal circulation^38^. However, the pathophysiological mechanisms underlying miR-125b and preeclampsia remains largely unclear.

As miRNAs usually play their roles via targeting mRNA transcripts, thus the exploration of miR-125b target genes is a key footstep for clarifying the pathophysiological significance of miR-25b in PE. We employed miRNA-target prediction algorithms (Targetscan, miRanda, and MirTarget) to screen putative targets of miR-125b and validated the predicted genes in the placenta of preeclamptic patients and control pregnancies. We found KCNA1 and GPC1 were significantly downregulated in placentas from preeclamptic patients. Correlation analysis suggested that miR-125b expression in the placenta was inversely correlated with the expression of KCNA1 and GPC1 in the placenta tissue, which made us more confident to conduct further exploration. Previous reports indicated that miR-125b localized in the trophoblast and endothelial cells^39^. To our surprise, when we perform immunohistochemistry to determine whether the putative target genes expressed in the placenta, both KCNA1 and GPC1 had positive signals in the placenta with different expression patterns, KCNA1 mainly expressed in trophoblast cells, and GPC1 predominantly observed in endothelial cells. In vitro cell assay revealed that miR-125b negatively regulated KCNA1 expression in HTR8/SVneo and GPC1 expression in HUVECs. We further validated the direct targeting effect of miR-125b in HEK-293T cells. The findings demonstrated that miR-125b significantly inhibited KCNA1 in trophoblast cells, and suppressed GPC1 expression in endothelial cells, suggesting KCNA1 and GPC1 may play distinct roles in the pathogenesis of preeclampsia.

We assessed the consequence of miR-125b upregulation in an immortalized first-trimester trophoblast cell line, HTR8/SVneo. We observed that trophoblast cells transfected miR-125b displayed a significant decrease in cell invasion, which is similar to KCNA1 knockdown by siRNA. Indeed, accumulating studies demonstrated that KCNA1, as a selective potassium channel protein in the repolarization of cell membrane, was closely correlated with cell invasion in many tumors^27,28,40^. We then carried out a rescue experiment by overexpressing KCNA1, the data indicated that KCNA1 significantly abolished the invasion-inhibitory effect of miR-125b. Taken together, these results supported that miR-125b inhibited the invasiveness of human trophoblast cells via KCNA1.

We also investigated the impact of miR-125b on endothelial cell function in HUVECs. Consistent with previous reports^41,42^, we found that miR-125b significantly impaired tube formation of HUVECs. Rescue experiment proved that GPC1 overexpression significantly ameliorates the effect of miR-125b on tube formation in HUVECs. Glypican-1 (GPC-1) is highly expressed on the cell membrane, mainly modulate FGF and VEGF signaling^43^, Previous investigations reported that tumors without GPC1 exhibited decreased expression of VEGF, and endothelial cells from GPC1 knockout mice did not migrate in response to VEGF^44^. Collectively, the findings demonstrated that miR-125b impaired endothelial function of human endothelial cells by directly targeting GPC1.

The development of PE was initiated by reduced uteroplacental perfusion resulting from abnormal trophoblast invasion and impaired spiral artery remodeling. Here we demonstrated that in early pregnancy, upregulated miR-125b disturbs trophoblast invasion and endothelial cell function. Although the exact cellular sources of the observed upregulation of miR-125b remain to be determined, miR-125b is reported to expressed in the placenta cells^39,45^, and the placenta should be one of the essential sources of maternal circulating miR-125b^38,46^, further supporting the conclusion that miR-125b participated in the initiation of PE.

To our knowledge, our study had advantages in the following field: (1) we found a novel upregulated circulating miR-125b in early pregnancy from patients who later developed preeclampsia; the aberrantly elevated expression of miR-125b reduced significantly after delivery. (2) we provided strong evidence supporting the link between upregulated miR-125b in early pregnancy and abnormal placentation in the early development of PE.

Our study also has several limitations. First, we did not have the resources to collect more samples and do a long-term follow-up, which would have provided a better predictive or diagnostic value of miR-125b. An independent large retrospective study should be planned to provide more status for our discovery. Second, we did not take in late-onset preeclampsia patients. It has been shown early-onset and late-onset preeclampsia has different etiologies^47^. Moreover, we cannot exclude that the elevated expression levels of miR-125b in early pregnancy was just a following consequence due to other risk factors of the syndrome. Therefore, for the future study of miR-125b function in PE, transgenic miR-125b animal models will be required which allow for placenta specific overexpression that reflects better the actual situation in human preeclamptic patients, which will help to address in more detail how aberrant miR-125b expression contributes to the pathogenesis of PE in vivo.

In conclusion, we demonstrated that plasma miR-125b is aberrantly upregulated in patients who later developed preeclampsia as early as the 12th-13th week of gestation, and the aberrant upregulation of miR-125b reduced significantly to near normal state after the delivery, indicating mIR-125b as a predictive marker for the disorder. We provided strong evidence that miR-125b exerts multiple modulating effects during placentation by targeting KCNA1 and GPC1. Our findings revealed that elevated miR-125b could inhibit trophoblast invasion and angiogenesis via KCNA1 and GPC1, thus lead to poor placentation, and contribute to the pathology of PE. Our studies provide novel insights into the mechanisms underlying miR-125b and PE. Further studies are required to confirm the potential significance of our findings.

## Materials and methods

### Sample collection

The nested case–control study was drawn from a prospective screening for adverse obstetric outcomes in women who were visiting for their initial routine hospital attendance in pregnancy at Affiliated Hospital of Weifang Medical University which was held between 12th to 13th week of gestation. Maternal characteristics, medical history, and clinical measurements were recorded, and blood samples were taken. Data on pregnancy outcomes were obtained from the hospital case records.

Pregnancy outcomes were diagnosed by guidelines of the International Society for the Study of Hypertension in Pregnancy (ISSHP)^48^. The samples were obtained from preeclamptic patients and gestational age-matched healthy pregnancies without any other complications during pregnancy, maternal characteristics and demographics were listed in Table S1. Placenta tissues were sampled randomly around the central part of the placenta within 1 h of cesarean section and stored in liquid nitrogen immediately until bench studies.

Ethics approval was granted by the Ethics Committee of Weifang Medical University (Reference: 2016/033). All experimental methods and the consent forms were performed in accordance with the relevant guidelines. Written consent was obtained from all of the study participants before taking part.

### Immunohistochemistry

Freshly placenta samples were fixed in 4% paraformaldehyde, embedded in paraffin. For immunohistochemistry, the sections at 5 µm were antigen-retrieved in citrate antigen retrieval solution (pH = 6.8) at 95 °C for 15 min before being stained with antibody against KCNA1 (Sigma, Shanghai, China) and GPC1 (Abcam, Shanghai, China) at 4 °C overnight. The sections were next incubated with corresponding HRP-conjugated secondary antibodies (Zhongshan Goldenbridge, Beijing, China) for 1 h at RT and were visualized with diaminobenzidine (Zhongshan Goldenbridge).

### Cell culture and transfection

The human extravillous trophoblast cell line HTR8/SVneo was a kind gift from Prof. Charles H. Graham at Queen’s University, Canada and was cultured in Gibco RPMI1640 medium (Life Technologies, CA, USA) supplemented with 10% fetal bovine serum (FBS). Cells were incubated under 5% CO_2_ at 37 °C and passaged at 1:5 every 5 days.

After cells proliferate to ~70% confluency, transfection of 100 nM KCNA1 siRNA (5′-UUAAACAUCGGUCAGGAGC-3′, Invitrogen, Shanghai, China; Genbank ID for KCNA1: NM_000217), GPC1 siRNA (5′-GGGACACGCUCACGGCCAATT-3′, Invitrogen, Shanghai, China; Genbank ID for GPC1: NM_002081.2) or miRNA duplexes with Lipofectamine 2000 (Invitrogen, CA, USA) was conducted according the manufacturer’s manual.

### RNA extraction and quantification

Total RNA was extracted from 200 µL of plasma and 25 mg of placental tissue preserved in RNAlater (Ambion, Austin, USA) using the miRcute Serum/Plasma miRNA Isolation Kit (TIANGEN, Shanghai, China) and MiniBEST Universal RNA Extraction Kit (Takara, Dalian, China) separately according to the manufacturer’s instructions. Synthetic *Caenorhabditis elegans* microRNA (Cel-miR-39, 5′-UCACCGGGUGUAAAUCAGCUUG-3′, 1 µL of 01 nM, Takara, Dalian, China) was spiked into the human plasma samples and was used as an internal control.

miRNAs were reverse transcribed by PrimeScript™ RT Master Mix kit (Takara, Dalian, China) and subsequently quantified using TB Green^®^ Premix Ex Taq™ II kit (Takara, Dalian, China) with an Applied Biosystems 7500Fast (PerkinElmer, Foster City, CA). KCNA1 and GPC1 expressions were detected by standard real-time qPCR reactions and were normalized to GAPDH. The nucleotide sequences of specific primers were listed in Table S2.

### Western blotting

Proteins were prepared using radioimmunoprecipitation assay buffer (RIPA) as previously reported^23^. Briefly, lysate protein concentrations were measured by BCA Assay (Thermo Fisher, USA). Protein extracts were separated by 10% SDS-PAGE and subsequently electro-transferred to the nitrocellulose membranes (GE Lifescience, USA). The membranes were blocked and then incubated with primary antibodies rabbit anti-KCNA1 (Sigma, Shanghai, China), rabbit anti-GPC1 (Abcam, Shanghai, China) and mouse anti-actin (Abcam, USA) overnight at 4 °C after 5% BSA block, and HRP-conjugated secondary antibodies (Invitrogen, CA, USA) were incubated at room temperature for 2 h at following day. Specific signals were examined using a Pierce Enhanced Chemiluminescence Plus kit (Life Technology, USA) and recorded with FluorChem Q (Proteinsimple, MD, USA). The band intensities were quantitated by Image J v1.50 (NIH, USA), relative densities of KCNA1 and GPC1 were normalized to actin of the same blot.

### Dual-Luciferase eporter Assay

The HEK-293T cells were co-transfected with 80 ng of pMIR-REPORT plasmid construct containing wild-type/mutant 3′-UTRs of KCNA1 or GPC1 and 50 nM of miR-125b mimics or negative controls. In all, 48 h later, luciferase activities were measured using Dual-Glo Luciferase Assay System (Promega) according to the manufacturer’s instructions. The experiments were repeated three times with triplicate in each group independently.

### In vitro Tranwell insert invasion assay

In vitro Transwell insert invasion assay was performed as previously described^49^. In brief, the human trophoblast HTR8/SVneo cells were seeded in 150 µg/ml matrigel-precoated Transwell inserts with 8 μm pores (Costar, Cambridge, MA). In all, 1 × 10^5^ cells per well were placed into the upper chamber in 200 μl serum-free RPMI 1640 media. In all, 800 μl of media with 10% FBS was seeded on the outside of transwell. 24 h later, the membranes were cleaned with PBS, fixed in 100% methanol and stained with hematoxylin. Stained cells were photographed of five random fields, and invaded cells were counted with Image J. The invasion index was calculated as a percentage of invaded cell number normalized to the control group. All experiments were repeated four independent times in triplicate.

### Tube formation

In all, 70% confluency HUVECs were 0.25% trypsinized and seeded onto 24-well plates that were coated with Matrigel (BD Bioscicence, USA) and cultured at 37 °C for 30 min. A total of 20,000 HUVECs transfected with miR-125b and pcGPC1 were suspended in 100 µL ECM (Sciencecell, USA) and seeded. After 6 h culturing, the endothelial tube-like structures were observed under an inverted microscope and images captured from five randomly selected microscopic fields. The tube length was measured and analyzed using Image J software (NIH, USA).

### Statistical analysis

Statistical analysis

All quantitative values were expressed as mean ± SEM based on ≥3 independently repeated experiments. Statistical comparisons between two groups were evaluated by the Student’s *t* test with SPSS 17.0 software (SPSS Inc., USA), and *p* < 0.05 were considered statistically significant. All graphical representations were produced using GraphPad Prism v7.0 software (GraphPad Software, CA, USA).

## Supplementary information

Supplmental Tables

Supplement Figure Legends

Figure S1

Figure S2

Figure S3

Figure S4

Figure S5

Figure S6

Figure S7
