# Supplementary material for: Elevated microRNA-125b inhibits cytotrophoblast invasion and impairs endothelial cell function in preeclampsia
Source: Cell Death Discov. 2020 May 13;6:35. doi: 10.1038/s41420-020-0269-0 (PMC7220944; doi:10.1038/s41420-020-0269-0)
Supplement: Supplementary file 1 — Supplmental Tables [file 41420_2020_269_MOESM1_ESM.docx]

**Table S1. Demographics and clinical characteristic of patients enrolled in current study.**

|  | Ctrl  (N=29) | PE  (N=15) | *p*-value |
| --- | --- | --- | --- |
| Age, y | 30.62 ± 0.72 | 31.13 ± 1.24 | 0.7042 |
| BMI, kg/m^2^ | 22.56 ± 0.61 | 22.74 ± 0.79 | 0.8610 |
| SBP, mm Hg | 110.9 ± 1.3 | 151.7 ± 3.5 | < 0.0001 |
| DBP, mm Hg | 76.2 ± 1.3 | 102.1 ± 2.7 | < 0.0001 |
| Proteinuria, g/24h | NA | 3.01 ± 0.55 | NA |
| 50g GCT, mM | 6.48 ± 0.27 | 6.65 ± 0.39 | 0.7185 |
| Gestation day at delivery, d | 271 ± 1.9 | 240 ± 1.7 | < 0.0001 |
| Infant birth weight, g | 3276 ± 81 | 2302 ± 136 | < 0.0001 |

Data are shown as mean ± SEM, and differences between normal and SPE patients were analyzed with *Student-t* test. BMI, body mass index; SBP, systolic blood pressure; DBP, diastolic blood pressure; GCT, glucose challenge test; NA, not available.

**Table S2 Sequences of specific primers used for Real-time qPCR**

| **Gene** | **Primer** | **Sequence 5’-3****’** |
| --- | --- | --- |
| KCNA1 | Forward | CACCGAGATAGCTGAGCAGG |
|  | Reverse | GGCGGGAGAGCTTGAAGATT |
| GPC1 | Forward | GAGGCTGGTGGCTGCTATG |
|  | Reverse | CTTGGCTCCGTAGATCTGGC |
| GAPDH | Forward | CTTCGCTCTCTGCTCCTCCTGTTCG |
|  | Reverse | ACCAGGCGCCCAATACGACCAAAT |
| miR-125b | Forward | TCCCTGAGACCCTAACTTGTGA |
| U6 | Forward | CGCTTCGGCAGCACATATAC |
|  | Reverse | AAAATATGGAACGCTTCACGA |
