# Supplementary material for: Elevated microRNA-125b inhibits cytotrophoblast invasion and impairs endothelial cell function in preeclampsia
Source: Cell Death Discov. 2020 May 13;6:35. doi: 10.1038/s41420-020-0269-0 (PMC7220944; doi:10.1038/s41420-020-0269-0)
Supplement: Supplementary file 2 — Supplement Figure Legends [file 41420_2020_269_MOESM2_ESM.docx]

**Figure S1. miR-125b is upregulated in preeclampsia.**

**(A)** Comparison of miR-125b mature sequence among hemochorial placenta species shows complete conservation, including the seeding region binding to 3’-UTRs of target transcripts. miR-125b levels were determined in the basal plates **(B)** and chorionic plates **(C)** of placentas derived from PE patients and compared to control pregnancies. **(D)** ROC analysis of plasma miR-125b. ROC curves revealed that plasma miR-125b in early pregnancy had a high accuracy in identifying PE patients before disease onset with an AUC of 0.7471 (95% CI, 0.5931-0.9012, *p*<0.01). **(E)**

**Figure S2. Inhibition of endogenous miR-125b increased KCNA1 expression in HTR8/SVneo cells.**

The human trophoblast cells, HTR8/SVneo, were transfected with miR-125b inhibitor or negative control oligonucleotides. mRNA levels **(A)** and protein levels **(B)** of KCNA1 was determined post transfection in using Real-time qPCR and Western blot separately. Data are presented as mean ± SEM of four independent experiments in triplicate. **p* < 0.05, ***p* < 0.01.

**Figure S3. Inhibition of endogenous miR-125b increased GPC1 expression in HUVECs.**

The human endothelial cells, HUVECs, were transfected with miR-125b inhibitor or corresponding negative control. 48 hours later, mRNA levels **(A)** and protein levels **(B)** of putative target GPC1 was tested using Real-time qPCR and Western blot separately. Data are presented as mean ± SEM, N=4 performed in triplicate. **p* < 0.05, ***p* < 0.01.

**Figure S4. Generation of luciferase reporter constructs containing 3’-UTRs of KCNA1 or GPC1.**

Sequences within the 3′-UTRs of KCNA1 and GPC1 containing putative binding sites for miR-125b that were cloned into the pMIR-REPORT vector to create wild-type or mutated (m) luciferase reporter plasmids. Sequences of the wild-type and mutated putative miR-125b binding sites within the 3′-UTRs of KCNA1 **(A)** and GPC1 **(B)** were presented. The mutated bases within the 3′-UTR of each mRNA transcript were indicated in red. (C) Schematic map of pMIR-REPORT^TM^ luciferase reporter constructs.

**Figure S5. Validation of KCNA1 overexpression of pcDNA4 plasmid overexpression KCNA1 (pcKCNA1).**

mRNA **(A)** and protein **(B)** level of KCNA1 in human trophoblast HTR8/SVneo cells transfected with pcKCNA1 vector or pcDNA4 plasmid were detected using Real-time qPCR and Western blot, separately. Data are presented as mean ± SEM of four independent experiments, each experiment had three parallel treatment. **p* < 0.05, ***p* < 0.01.

**Figure S6. overexpression efficacy of pcDNA4 plasmid overexpression GPC1 (pcGPC1).**

mRNA **(A)** and protein **(B)** level of GPC1 in human endothelial cells, HUVECs, transfected with pcKCNA1 vector or pcDNA4 plasmid were examined using Real-time qPCR and Western blot, separately. Data are presented as mean ± SEM of four independent experiments in triplicate. **p* < 0.05, ***p* < 0.01.

**Figure S7. Typical image of the negative control slides in immunohistochemistry.**

Negative control of immunohistochemistry was performed on paraffin sections of human placenta villi. The slides were incubated with rabbit pre-IgG overnight and then HRP-conjunct secondary antiboy.
