# Supplementary figures and images for: Elevated microRNA-125b inhibits cytotrophoblast invasion and impairs endothelial cell function in preeclampsia
Source: Cell Death Discov. 2020 May 13;6:35. doi: 10.1038/s41420-020-0269-0 (PMC7220944; doi:10.1038/s41420-020-0269-0)

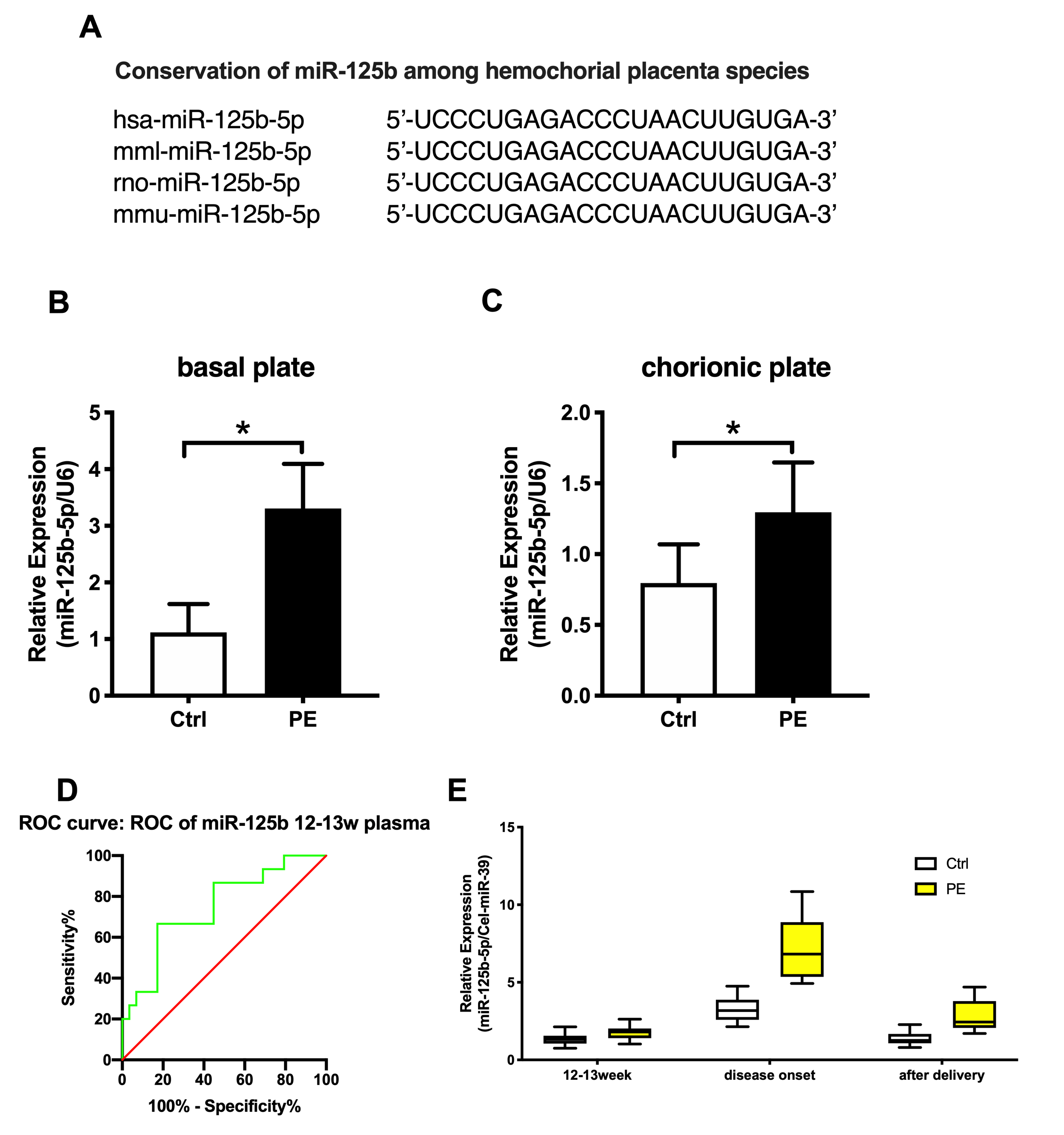

Supplement: Supplementary file 3 — Figure S1 [file 41420_2020_269_MOESM3_ESM.tif]

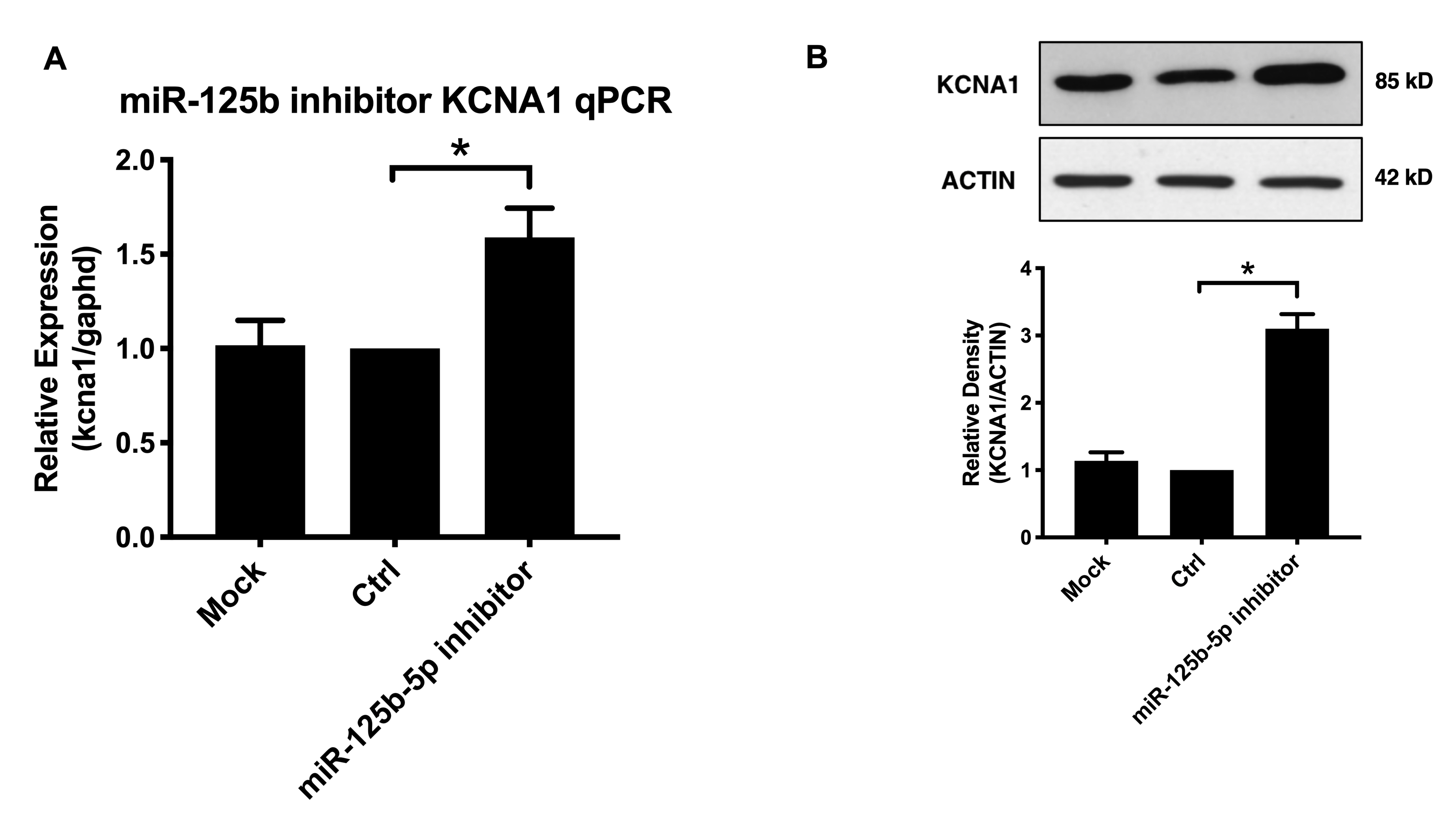

Supplement: Supplementary file 4 — Figure S2 [file 41420_2020_269_MOESM4_ESM.tif]

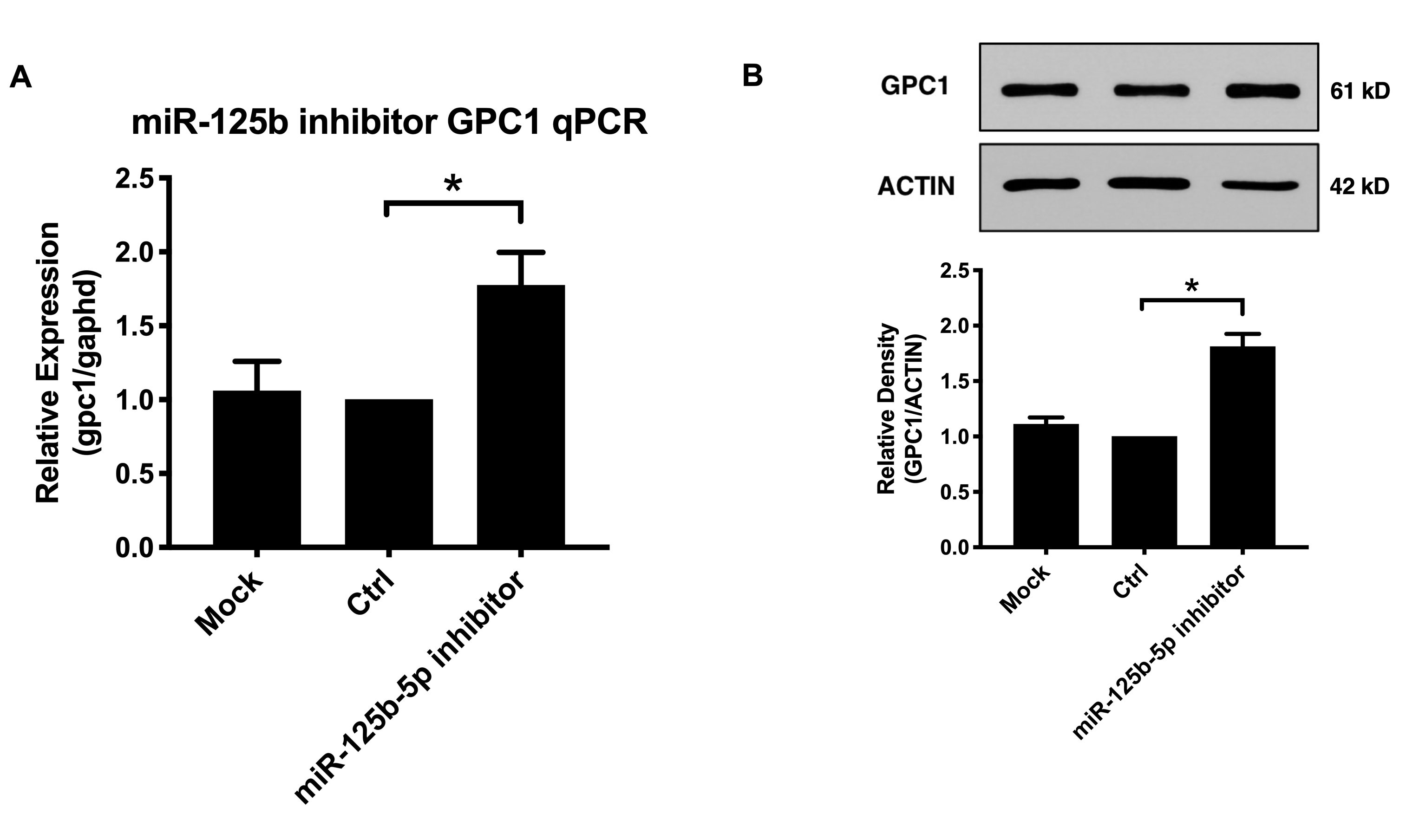

Supplement: Supplementary file 5 — Figure S3 [file 41420_2020_269_MOESM5_ESM.tif]

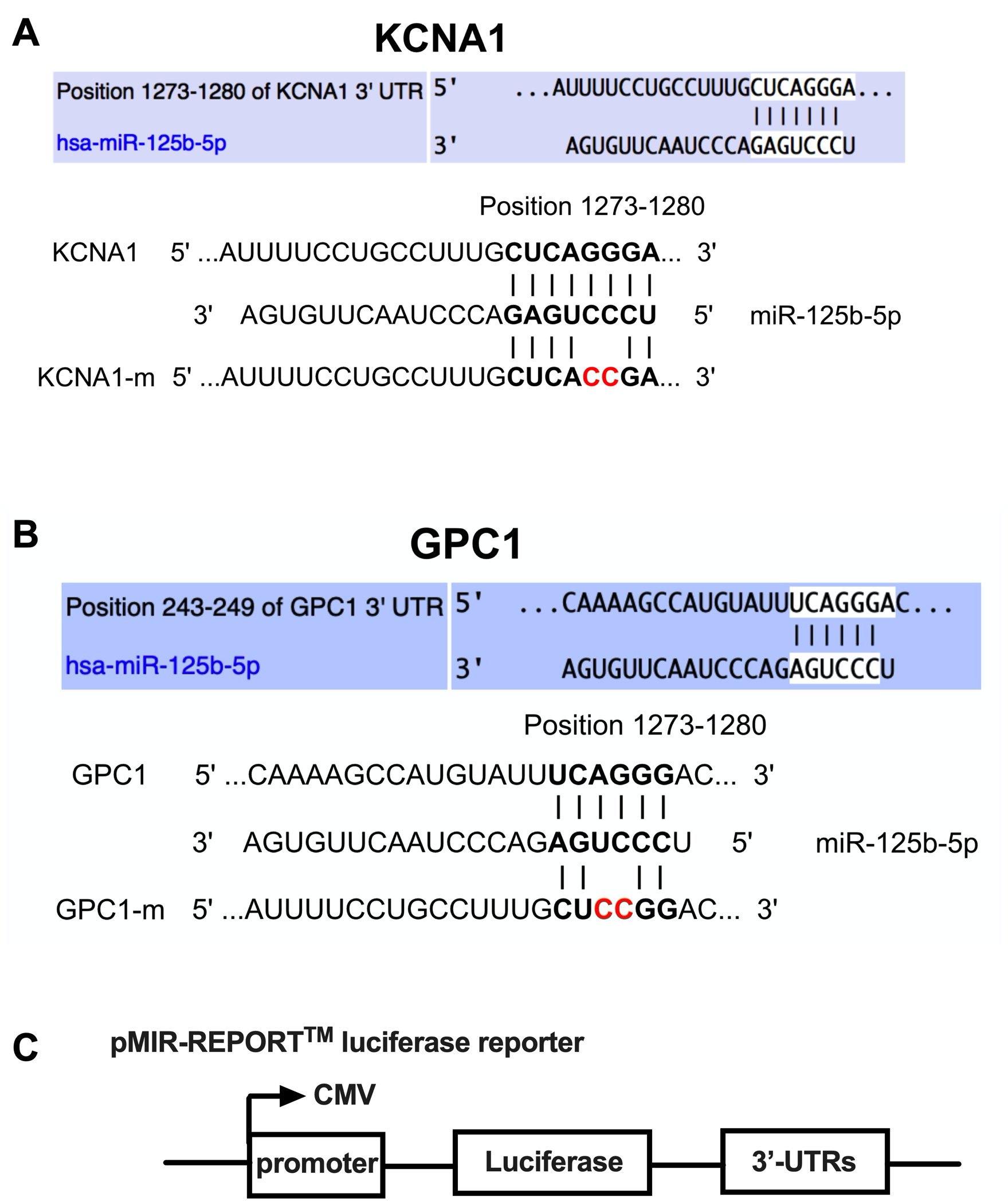

Supplement: Supplementary file 6 — Figure S4 [file 41420_2020_269_MOESM6_ESM.tif]

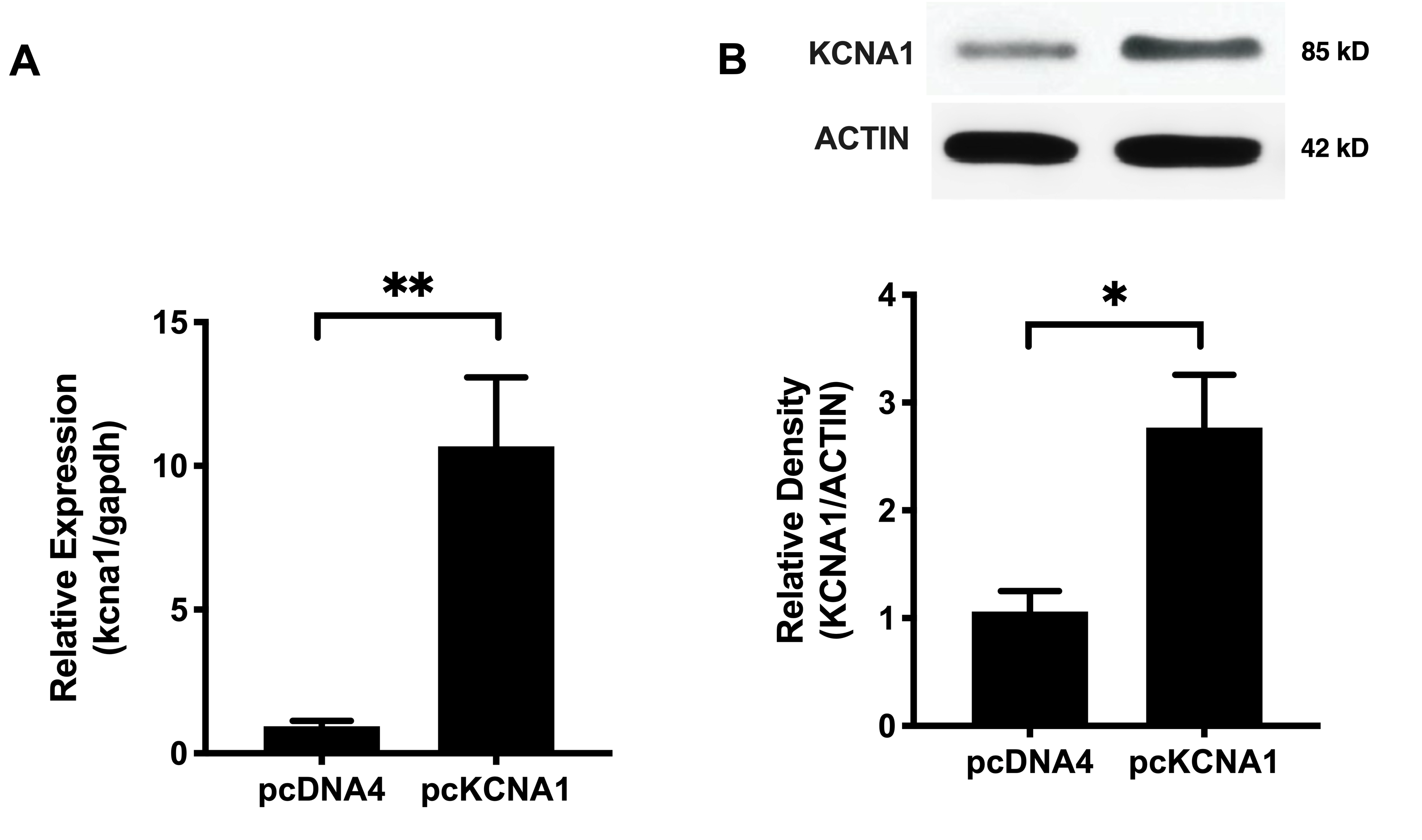

Supplement: Supplementary file 7 — Figure S5 [file 41420_2020_269_MOESM7_ESM.tif]

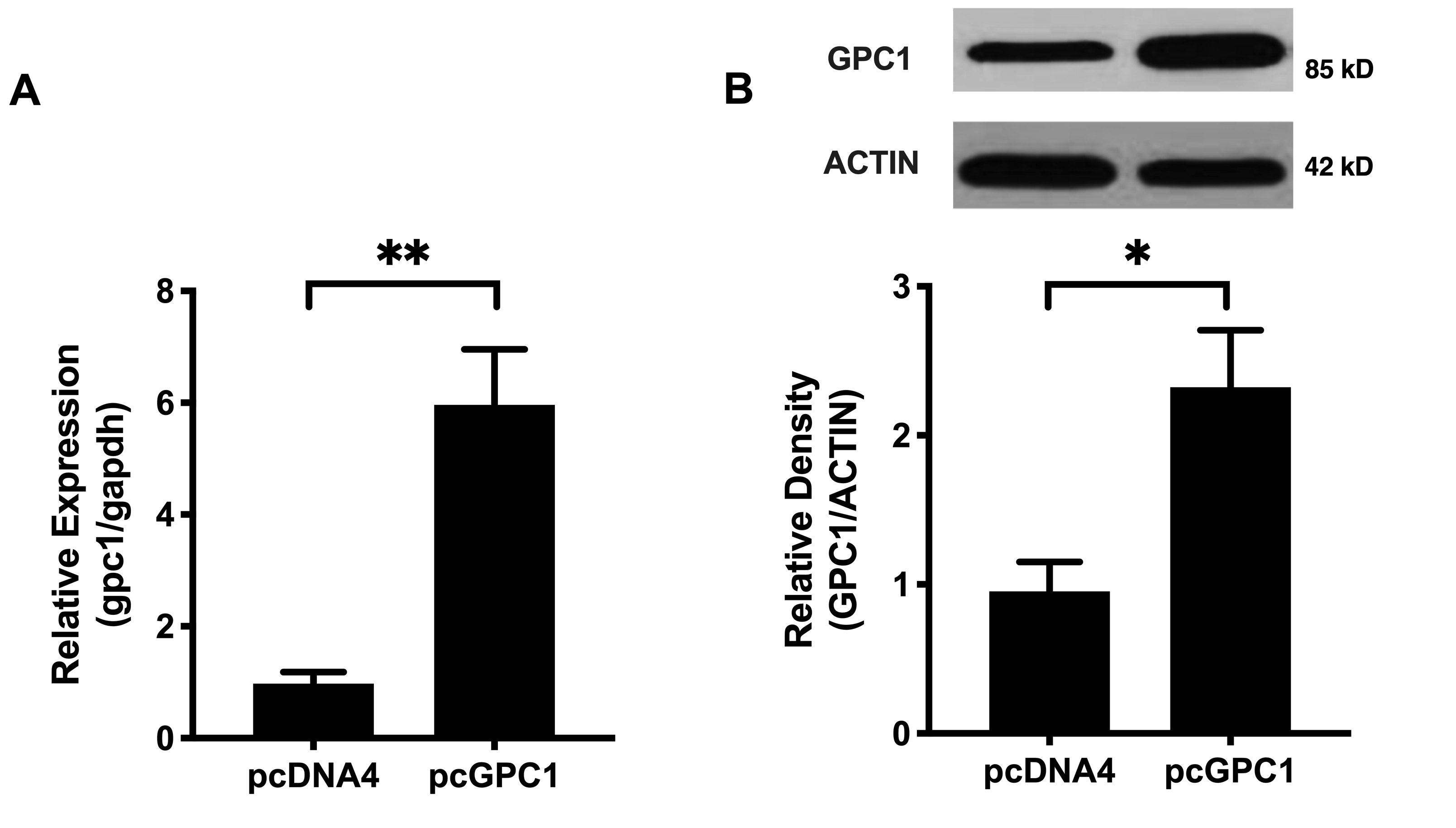

Supplement: Supplementary file 8 — Figure S6 [file 41420_2020_269_MOESM8_ESM.tif]

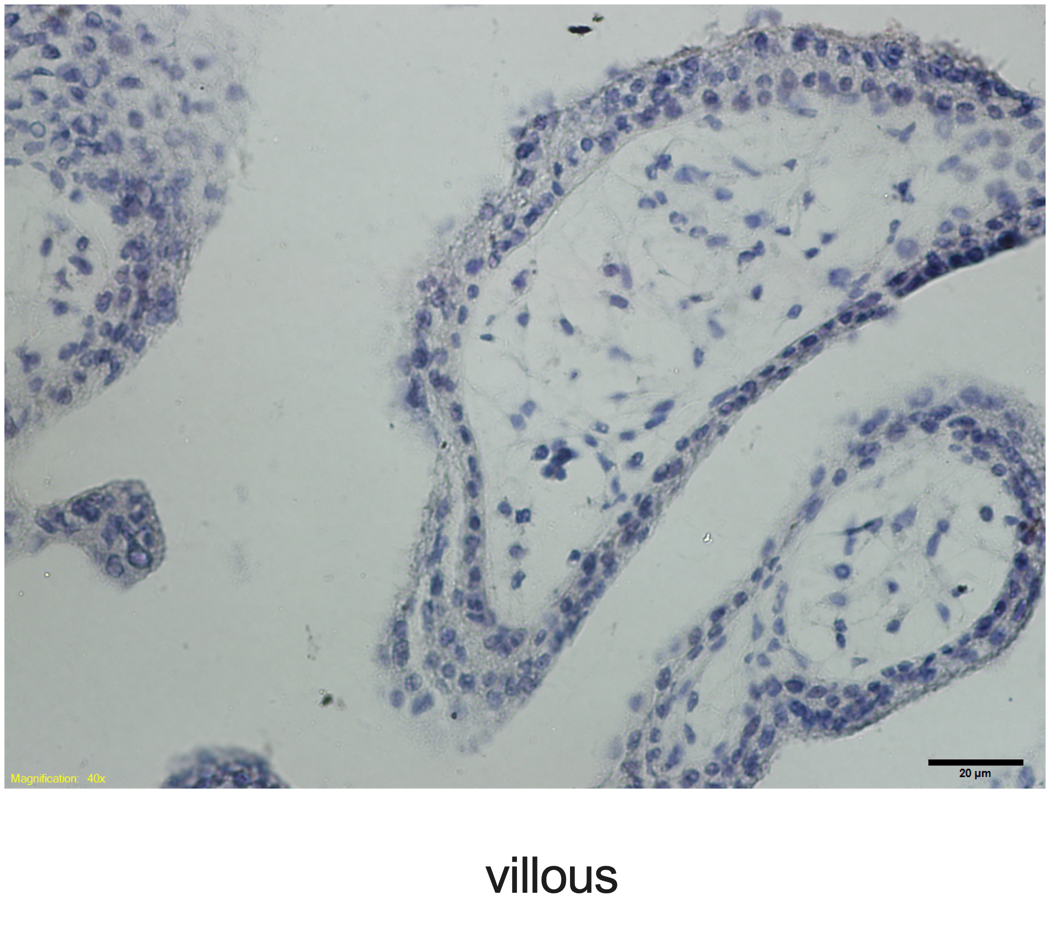

Supplement: Supplementary file 9 — Figure S7 [file 41420_2020_269_MOESM9_ESM.tif]
